# News and Coming Events

**Published:** 1907-02-09

**Authors:** 


### NEWS AND COMING EVENTS.

THE Hunterian Oration at the Royal College of Surgeons will be delivered on Thursday, February 14, at four o'clock in the afternoon. The orator for the year is Mr. Henry T. Butlin, F.R.C.S., D.C.L., consulting surgeon to St. Bartholomew's Hospital.

IN compliance with a wish of the popular actor, the executors of the late Mr. J. H. Toole have endowed a bed in Charing Cross Hospital to be known as the Toole bed. A further sum of £225 has been left by Mr. Toole to the National Hospital for the Paralysed and Epileptic, Queen Square.

THE next meeting of the University College Medical Society will be held in the Medical Library, University College, Gower Street, on Wednesday, February 13, at 8.30. Dr. Risien Russell will be in the chair, and Dr. Henry Head, F.R.S., will read a paper on "The Grouping of Sensory Impulses."

A CONSTANTINOPLE correspondent reports that the Ottoman Government has decided to build a new hospital at Stamboul as an annexe to the Hamadié Hospital at Shishli. The new building will have a special ward for obstetric and gynæcological cases, and among the private subscribers to the building fund is H.I.M. the Sultan.

THE Royal Sanitary Institute will meet at Stafford on February 18, opening its proceedings with a paper for discussion by Dr. Reid, medical officer of health for the county, on Sewage Purification. Medical men desirous of attending the lunch and opening meeting are requested to apply direct to the secretary not later than February 9.

THE second Lettsomian Lecture before the Medical Society of London will be delivered on February 18; the third will be delivered on March 9. The lectures commence at 9 P.M., and the lecturer, Dr. C. E. Beevor, has chosen as his subject the many interesting points in connection with the diagnosis and localisation of cerebral tumours.

THE death is reported of Professor Budin who died at Marseilles during the latter end of January from an attack of pneumonia. Budin succeeded Tarnier as teacher of clinical obstetrics in the University of Paris, and his work in gynæcological and obstetrical medicine has become classical. His latest work, "The Nursling," of which an English translation has just been published, has had a wide popularity in France. Professor Budin was an enthusiastic member of the League Against Infantile Mortality, of which he was one of the founders, and his last public appearance was at the annual conference of the League.

AN interesting report has been issued by the Commission recently appointed by the Scottish Temperance Legislation Board to visit Norway and report upon the liquor licensing laws there in force. The report gives a full account of the existing legislation in Norway with reference to the sale and consumption of intoxicants. Local option is exercised very largely, and total prohibition is favoured in the smaller towns; the larger towns preferring "stringent control and restriction." Such control is vested in the "Samlags"—disinterested companies of management. The commissioners put on record their opinion that this limitation of the liquor traffic has done much to transform Norway from one of the most drunken to one of the most sober of European nations.

AMONG the more important forthcoming meetings of foreign professional societies are the following: the German Society of Orthopædic Surgery, sixth annual congress, at Berlin on April 2; the German Surgical Society, annual meeting at Berlin on the same date; the Seventh International Congress of Physiology, at Heidelberg, commencing August 13, under the presidency of Professor Kossel; German Balneological Association, eighth annual congress under the chairmanship of Professor Liebreich, at Berlin on March 7.

THE annual meeting of the subscribers of the Italian Hospital, Queen Square, W.C., was held on January 18, the Marchese di San Guiliano, the Italian Ambassador, presiding. This hospital has treated more than 17,000 patients during the year. In moving the adoption of the report of the hospital committee Dr. Cassaneda, the doyen of the consulting staff, stated that the King of Italy was cordially in agreement with the committee's proposal to establish an endowment fund for the hospital.

THE Secretary of the Rural Housing and Sanitation Association makes an appeal on behalf of an Association which has done, and is doing, very useful work. The Rural Housing and Sanitation Association is entirely non-political. It was founded some four years ago, and has already been the means of improving sanitation and increasing and improving the supply of cottages in many country villages. The importance of such work, not only in raising the health of the population, but in helping to stem the tide of migration to the towns, can hardly be over-estimated; and in those cases where the authorities are apathetic or local interests are hostile, the assistance of an independent association with experience of other cases to guide it has been found to be of the greatest possible value. Everywhere it is useful in forming public opinion and strengthening the hands of those who are working for moderate measures of reform. Subscriptions and donations may be sent to the Secretary, at the offices of the Association, Parliament Mansions, Victoria Street, S.W.

AN important Army Order was issued on Saturday last with reference to the instruction of officers, non-commissioned officers, and men in sanitation. It is laid down that general officers commanding-in-chief shall arrange for at least one annual course of lectures in sanitation for officers, the lectures to be given by the command sanitary officer or by a selected officer of the Royal Army Medical Corps. After March 1, 1908, lieutenants will be required to pass an examination in sanitation before promotion to the rank of captain, and officers of companies, squadrons, etc., will give instruction to their non-commissioned officers and men in sanitation. On mobilisation being ordered, a Sanitary Inspection Committee will be formed for service in the field. The duties of the Committee will be to ascertain that sanitary appliances and materials of all kinds required for the Army are forthcoming, and that an adequate reserve is maintained; to assist general officers and the Medical Service in their efforts to maintain the health of the Army by co-ordinating not only the work of the different military branches, but also the military and the civil organisations of the country or area occupied; to initiate schemes of general sanitation, and to serve as a board of reference for the solution of sanitary questions; to visit and inspect stations occupied by troops, to advise local authorities regarding necessary sanitary measures, and to further in every way the maintenance of satisfactory sanitary conditions.

